# Pure laparoscopic major liver resection after yttrium^90^ radioembolization: a case-matched series analysis of feasibility and outcomes

**DOI:** 10.1007/s00423-022-02474-z

**Published:** 2022-02-28

**Authors:** Daniel Aliseda, Pablo Martí-Cruchaga, Gabriel Zozaya, Alberto Benito, Luis Lopez-Olaondo, Macarena Rodríguez-Fraile, José I. Bilbao, Francisco Hidalgo, Mercedes Iñarrairaegui, Rubén Ciria, Fernando Pardo, Bruno Sangro, Fernando Rotellar

**Affiliations:** 1 HPB and Liver Transplant Unit, Department of General Surgery, Clinica Universidad de Navarra, University of Navarra, Av. Pío XII, 36, 31008 Pamplona, Spain; 2 Institute of Health Research of Navarra (IdisNA), Pamplona, Spain; 3 Interventional Radiology, Department of Radiology, Clinica Universidad de Navarra, University of Navarra, Av. Pío XII, 36, 31008 Pamplona, Spain; 4 Anesthesiology Unit, Clínica Universidad de Navarra, University of Navarra, Av. Pío XII, 36, 31008 Pamplona, Spain; 5 Nuclear Medicine Department, Clinica Universidad de Navarra, University of Navarra, Av. Pío XII, 36, 31008 Pamplona, Spain; 6 Liver Unit and HPB Oncology Area, Clinica Universidad de Navarra and CIBEREHD, Pamplona, Spain; 7 Unit of Hepatobiliary Surgery and Liver Transplantation, IMIBIC, University Hospital Reina Sofía, University of Cordoba, Cordoba, Spain

**Keywords:** Laparoscopic liver resection, Radioembolization, Hepatocellular carcinoma, Colorectal liver metastases, Intrahepatic cholangiocarcinoma

## Abstract

**Background:**

Liver surgery after radioembolization (RE) entails highly demanding and challenging procedures due to the frequent combination of large tumors, severe RE-related adhesions, and the necessity of conducting major hepatectomies. Laparoscopic liver resection (LLR) and its associated advantages could provide benefits, as yet unreported, to these patients. The current study evaluated feasibility, morbidity, mortality, and survival outcomes for major laparoscopic liver resection after radioembolization.

**Material and methods:**

In this retrospective, single-center study patients diagnosed with hepatocellular carcinoma, intrahepatic cholangiocarcinoma or metastases from colorectal cancer undergoing major laparoscopic hepatectomy after RE were identified from institutional databases. They were matched (1:2) on several pre-operative characteristics to a group of patients that underwent major LLR for the same malignancies during the same period but without previous RE.

**Results:**

From March 2011 to November 2020, 9 patients underwent a major LLR after RE. No differences were observed in intraoperative blood loss (50 vs. 150 ml; *p* = 0.621), operative time (478 vs. 407 min; *p* = 0.135) or pedicle clamping time (90.5 vs 74 min; *p* = 0.133) between the post-RE LLR and the matched group. Similarly, no differences were observed on hospital stay (median 3 vs. 4 days; *p* = 0.300), Clavien–Dindo ≥ III complications (2 vs. 1 cases; *p* = 0.250), specific liver morbidity (1 vs. 1 case *p* = 1.000), or 90 day mortality (0 vs. 0; *p* = 1.000).

**Conclusion:**

The laparoscopic approach for post radioembolization patients may be a feasible and safe procedure with excellent surgical and oncological outcomes and meets the current standards for laparoscopic liver resections. Further studies with larger series are needed to confirm the results herein presented.

**Supplementary Information:**

The online version contains supplementary material available at 10.1007/s00423-022-02474-z.

## Introduction

Radioembolization (RE), also known as selective internal radiation therapy, is a liver-directed therapy that is based on transarterial delivery of high-dose beta radiation to the tumor-associated capillaries, thereby sparing healthy liver tissue [1]. Recent clinical experience suggests that patients with liver-limited, unresectable disease may benefit from this therapy.

For patients with hepatocellular carcinoma (HCC) and intrahepatic cholangiocarcinoma (ICC), the published literature has shown that RE produces clinically significant reductions in tumor size leading to a downstaging that may allow, in some cases, access to a surgical approach with curative intent [2, 3]. It has also been reported that RE combined with chemotherapy may be a rescue procedure for initially unresectable colorectal liver metastases (CRLM), making them resectable in some selected cases [4]. Besides reducing tumor size, when injected into a lobar artery, RE produces contralateral hypertrophy [5]. This phenomenon is associated with a sustained increase in circulating levels of pro-regenerative factors [6]. This unique RE feature has also been reported to allow curative surgical intention in cirrhotic livers with previous insufficient future liver remnant (FLR) [7].

The local hepatic response to RE may generate severe adhesions to surrounding structures and induce hepatic fibrotic changes, which may lead to difficulties during the surgery [8, 9]. In 2009, Gulec et al. [10] published the first major hepatic resection after RE. In the years that followed, the first case series of liver resection after RE for HCC and CRLM appeared in the literature [3, 11]. However, perioperative surgical outcomes after RE were unknown until the multicenter Post-SIR-Spheres Surgery Study (P4S) [12] reported the adequate safety of open resection and transplantation after RE. Various articles have been published afterwards showing the outcomes of liver resection after RE [13, 14]. Although laparoscopic liver resections (LLRs) have become increasingly widespread worldwide with an increasing number of reported cases, only a few preliminary results from our own experience of LLR post-RE have been reported [15, 16].

The main aim of this observational, retrospective study is to describe the feasibility, short- and long-term outcomes, for pure major laparoscopic liver resection post-RE (MLLR-RE). The secondary aims are to compare these results with a case-matched cohort of patients undergoing major laparoscopic liver resection (Ct-MLLR) for the same hepatic malignancies but without previous RE, and to compare the results of the MLLR-RE series with the recently defined textbook outcomes for laparoscopic major liver surgery (TOLS)[17].

## Material and methods

All patients who underwent LLR for malignancies, including HCC, CRLM, or ICC with at least one prior RE treatment, from March 2011 to November 2020, and with a minimum 90-day follow-up were retrospectively analyzed from a prospectively maintained database and were included in the study. All patients provided written consent for the treatment. This study was performed following the Declaration of Helsinki ethical standards and was approved by the Institutional Review Board (2021.056).

### Patient evaluation and eligibility

All patients were evaluated by our institutional hepatopancreato-biliary malignancies multidisciplinary team (MDT). The indication for RE and the decision on whether to treat only the tumor—to obtain downsizing—or involve the entire lobe in the targeted volume to induce simultaneous hypertrophy of the FLR was considered by the MDT on a case-by-case basis.

There is rarely a unique reason for indicating RE, but usually a combination of several: need for local control or tumor size reduction, with or without need for hypertrophy of the future remnant (due to expected post-surgical FLR < 30% or < 40% in cirrhotic patients or those previously treated with chemotherapy) and always in a context of high biological risk that makes it advisable to provide a test of time in order to optimize the indication for surgery [3, 7].

Patients were only considered for RE if they had an Eastern Cooperative Oncology Group performance status not higher than 2, as well as preserved liver (absence of ascites and serum total bilirubin < 2 mg/dL), hematological (platelet count > 40/pL), and renal function (serum creatinine < 2 mg/dL), no contraindication to angiography, and were able to provide informed consent [18].

Patients who do not meet these criteria were considered unsuitable for RE. Additionally, the following conditions were considered exclusion criteria:

1. A lung shunt fraction > 20%
2. Previous stereotactic body radiation therapy to the liver
3. Presence of collateral vessels feeding extrahepatic organs

During the study period, the laparoscopic approach was the approach of choice in all patients, and no inclusion criteria were applied. The only exclusion criteria for rejecting the laparoscopic approach were those cases that required complex vascular management.

### Radioembolization protocol

All patients were treated according to our previously reported protocol for RE [18]. In brief, patients underwent an angiographic evaluation to identify possible accessory arteries feeding the tumors and to detect and embolize any collateral vessel feeding extrahepatic organs. Once the ideal site(s) for microsphere injection was identified, macroaggregated albumin (^99m^Tc-MAA) was injected and a SPECT-CT scan was performed to measure hepatopulmonary shunting, further detect any unnoticed shunting to other extrahepatic organs, and allow the determination of the tumor to non-tumor ratio that is needed to calculate yttrium-90 (^90^Y) activity [18]. Radioembolization was delivered with ^90^Y resin microspheres (SIR-Spheres; Sirtex Medical) (15). In all cases, a same-day calibration 3 GBq vial was used (44 ± 2.6 million spheres per vial) [19].

Toxicities observed between RE and surgery were classified according to the National Cancer Institute Common Terminology Criteria for Adverse Events v5 [20]. Surgery was indicated after ensuring response or stabilization of the hepatic tumor, absence of extrahepatic progression, and sufficient FLR.

### Volumetric assessment

Computed tomography or magnetic resonance imaging were performed at T0 (immediately before RE imaging) and T1 (2–6 months after RE). Tumor volume and hypertrophy were assessed as absolute (cm^3^) and relative (%) changes between volumes at T0 and at T1. Future liver remnant was defined as the ratio between the volume of the remnant liver after the planned surgery and total liver volume. The FLR was calculated at T0 and T1.

### Case-matched study design and endpoints

A case-matched study was performed to compare the 2 groups: pure MLLR-RE vs. a matched group who underwent Ct-MLLR without preoperative RE. A 1:2 ratio was designed; for any patient in the MLLR-RE, two Ct-MLLR patients operated on for the same hepatic malignancies and during the same period were matched by age, gender, body mass index (BMI), American Society of Anesthesiologists (ASA) score, IWATE score [21], pre-operative tumor size, cirrhosis, and operative procedure. Surgical safety, morbidity, and mortality were the main endpoints of this study. In order to ensure that the laparoscopic techniques/approaches and the learning curve (qualification) of the surgeon were homogeneous and equally distributed among all patients, we confirmed that all surgeries (RE and control group) were performed by the same surgeon, in the same period and distribution.

### Operative and oncological outcomes

All procedures, in both groups, were performed by the same HPB surgeon. Intraoperative parameters including operative time, intraoperative blood loss, transfusion, and clamping time as well as postoperative outcomes including hospital course, morbimortality (according to the Clavien–Dindo classification [22]), pathologic characteristics, and long-term oncological outcomes were prospectively collected. The IWATE score and the risk scoring system proposed by Halls et al. were used to determine the laparoscopic liver resection difficulty level and the risk of intraoperative adverse events [21, 23]. Post-hepatectomy liver failure (PHLF) and post-hepatectomy hemorrhage (PHH) were evaluated using the criteria of the International Group for the Study of Liver Surgery [24, 25]. Liver-specific complications were determined as biliary leakage, biliary stricture, ascites, and liver abscess formation around the remnant liver.

#### Pure major laparoscopic liver resection (surgical procedure)

The technical aspects for MLLR in our center have been published previously [15, 26, 27] and are outlined below. The patient is placed with legs apart, with the main surgeon standing between them. A pneumoperitoneum is created with a Veress needle and set at 12 mm Hg. An extracorporeal tourniquet is routinely placed around the hepatic pedicle [28] and the transection is performed under intermittent 15-min clamping and 5-min release ischemia. Our method for right hemiliver mobilization and the hanging maneuver in RH has been described elsewhere [29]. When oncologically safe (no proximity of tumor to the hilum), a Glissonian pedicle approach is preferred [26, 30, 31], performed extrahepatically or intraparenchymally depending on the case circumstances; otherwise, an intrafascial approach is adopted [26]. Ischemic demarcation (or the ICG counterstaining method since 2013) is used to define the transection line on the liver surface. In both LH and RH, the middle hepatic vein (MHV) is used as a landmark reference, with vein-guided surgery being performed on one side or the other of the MHV depending on whether it is to be preserved or resected [15, 26, 27]. In recent years, with a better understanding of the laparoscopic view, a caudal approach to the MHV has been proved to be a helpful strategy [26]. Intraoperative ultrasound is routinely used to assess the tumor, rule out other lesions, and guide the transection, particularly in demanding extended resections. Transection is performed with an alternating combination of CUSA and Ligasure, optimizing hemostasis with bipolar forceps and occasionally barbed sutures. The combination of a low CVP/intrathoracic pressure and the Pringle maneuver ensures a bloodless field and allows for meticulous progression. The specimen is regularly introduced into a retrieval bag and removed through a suprapubic incision. Once this incision is closed, the pneumoperitoneum is re-established, hemostasis checked, and a hemostatic/sealing material (Tachosil) placed on the transected surface. No drains are routinely left in place.

### Statistical analysis

Quantitative and categorical variables are described by median-range and number-percentage, respectively. After checking for normality with Shapiro–Wilk tests, continuous variables were compared by the Mann–Whitney *U* test and categorical variables were compared by Fisher’s exact test. Overall survival (OS) and disease-free survival (DFS) were calculated from the date of liver resection until death or recurrence/metastases respectively. Patient OS and DFS were estimated using the Kaplan–Meier method. A *p* value < 0.05 was considered to define statistical significance. Statistical analyses were conducted using STATA version 16 (StataCorp, College Station, Texas 77,845 USA).

## Results

**Fig. 1:**
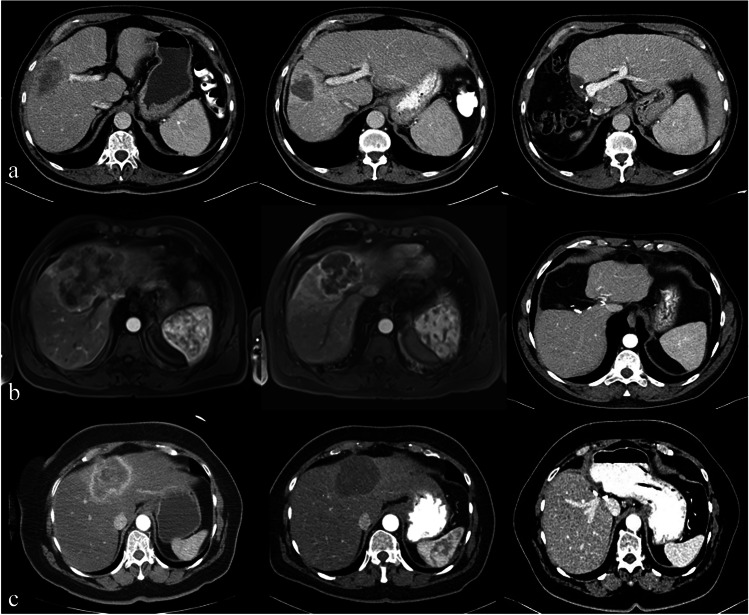
Pre radioembolization, pre-surgical, and post-surgical radiological exam (from left to right). **a** Right hepatectomy. **b** Central hepatectomy. **c** Extended left hepatectomy. Respectively patients 2, 7, and 8 in Table 1

During the study period, 21 consecutive patients, operated by the same HPB surgeon, underwent liver resections after RE in our Center. Nine patients received major laparoscopic resection and were included in the study (MLLR-RE). Of the remaining 12 cases, eight patients underwent major open liver resections and the remaining 4 underwent minor resections (3 laparoscopic resections and one open resection). The patients were 6 males and 3 females. Their median age was 67 years (46–74). Three patients had HCC, 3 ICC, and the remaining 3 had CRLM. The median pre-operative tumor size was 6.6 cm (range 3–16 cm). All HCC patients had underlying chronic liver disease which consisted of a cirrhosis grade Child–Pugh B7 (1 patient) and a biopsy-confirmed nonalcoholic steatohepatitis (2 patients). The pre-surgical profile of the MLLR-RE patients is shown in Table 1. The radiological tumor response and hypertrophy of the contralateral lobe for the three patients who underwent right, left, and central hepatectomy are shown in Fig. 1.

**Table 1:** Characteristics of the MLLR-RE group

Patient	Tumor type	Prior liver treatment	Comorbidities	Chemotherapy pre-RE	Chemotherapy post-RE	Prior abdominal surgery	RE to surgery (months)	IWATE score [21]	Operative procedure	Hospital stay (days)	Intra-/postoperative RBC transfusion	Complications (Clavien–Dindo ≥ III)
1	HCC	No	AHT, DM-II	None	None	None	3	11	LRH	3	0/0	none
2	ICC	Ablation	AHT	None	None	None	5	11	LRH	3	0/0	none
3	CRLM	Resection	None	≥ 2 line	≥ 1 line ^a^	Open Hartmann’s procedure Hepatic II-III laparoscopic tumorectomy	4	10	LRH	2	0/0	none
4	HCC	No	AHT	None	None	Open cholecystectomy Open appendicectomy Laparoscopic partial colon resection	8	10	LRH	11	0/0	Reoperation due to unnoticed small bowel perforation (IIIb)
5	ICC	No	AHT, OB	1 line	≥ 1 line	Laparoscopic cholecystectomy	3	10	LRH	5 + 10	2/0	Readmission due to infected biloma (IIIa)
6	HCC	No	AHT, CP, OB	None	None	Laparoscopic radical prostatectomy Laparoscopic ureterectomy	4	10	LLH	4	0/0	none
7	CRLM	No	None	1 line	≥ 1 line	Laparoscopic low anterior rectum resection	9	11	LCH	3	0/0	none
8	ICC	No	AHT	1 line	≥ 1 line	Open cholecystectomy Open appendicectomy	4	9	E-LLH	3	0/0	none
9	CRLM	Ablation	AF	1 line	≥ 1 line	Diagnostic laparoscopy Cytoreductive surgery	6	8	E-LLH	2	0/0	none

*HCC* hepatocellular carcinoma, *ICC* intrahepatic cholangiocarcinoma, *CRLM* colorectal liver metastasis, *BMI* body mass index, *ASA* American Society of anesthesiologist, *CP* cardiopathy, *AHT* arterial hypertension, *DM-II* diabetes mellitus II, *OB* obesity, *AF* atrial fibrillation, *MLLR-RE* major laparoscopic liver resection post radioembolization group, *RE* radioembolization, *LRH* laparoscopic right hepatectomy, *LLH* laparoscopic left hepatectomy, *LCE* laparoscopic central hepatectomy, *E-LLH* extended left hepatectomy, *RBC* red blood cells

^a^Dendritic cell immunotherapy

### Radioembolization characteristics

All patients received a single RE procedure with a median time from RE to surgery of 4 months (range 3–9 months). Median ^90^Y activity was 1.5 GBq (range 1–2 GBq). Lobar RE was performed in 5 patients (left lobe in 3 patients and right lobe in 2 patients), lobar extended RE in 1 patient (left lobe + segment VII), and segmental RE in 3 patients. No patient received radiation to the whole liver. No patients presented complications after RE as defined by the National Cancer Institute Common Terminology Criteria for Adverse Events v5.0 [20].

### Volumetric changes and tumor response

**Fig. 2:**
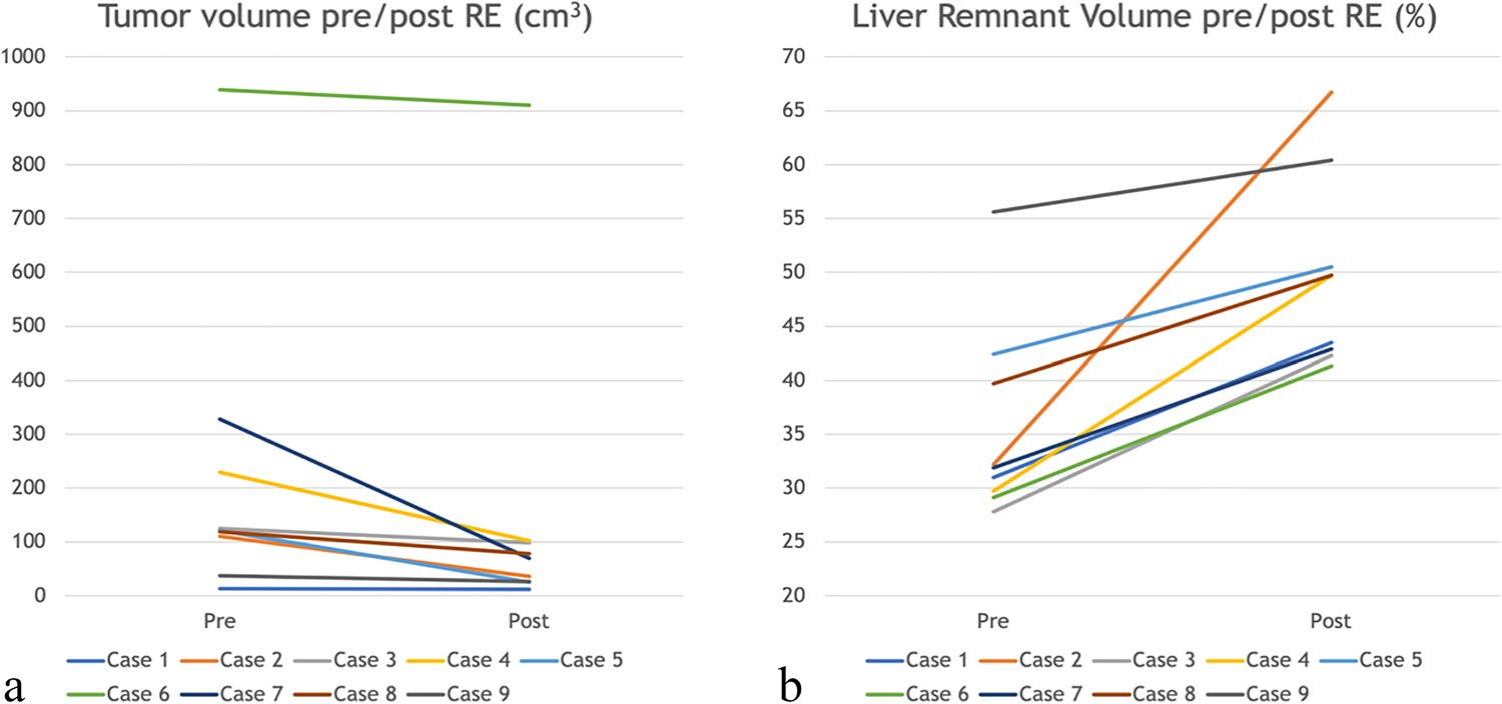
**a**, **b** Tumor and future liver remnant volumetric changes caused by RE

An increase in the non-treated liver volume with a consequent increase in FLR was observed in all patients. Five patients (55.5%) were considered non-candidates for surgery at diagnosis due to insufficient FLR. The indications for which we can apply RE prior to surgery are local control of the disease/reduction of tumor size with or without need of hypertrophy of the future liver remnant. These three aspects are local characteristics within a context of high biological tumor risk. Median FLR was 31.9% at T0 (range 27.8–55.6%) and 49.7% (41.3–66.7%) at T1. Future liver remnant percentage increased a median of 12.2% (4.8–34.8%) between T0 and T1. Consequently, after RE, all patients had sufficient FLR to undergo major hepatectomy. Median tumor volume at T0 was 120.8 cm^3^ (13.6–939.3). Tumor response was observed in varying degrees in each patient with an absolute and relative median tumor reduction of 41.4 cm^3^ (1.9–258.1) and 34.8% (3–79.5), respectively. Volumetric changes are graphically shown in Fig. 2.

### Surgical outcomes of M-LLR after RE

Surgical and post-operative aspects are shown in Table 1 (individual) and Table 2 (MLLR-RE matched analysis). All procedures were completed under a purely laparoscopic approach. In this MLLR-RE cohort, 9 major resections were completed: five right hepatectomies (two required partial diaphragmatic resection and suture due to severe RE-related liver adhesions), one left hepatectomy, two left extended hepatectomies (Fig. 4) (one of those—due to ICC—with regional lymphadenectomy and extended to the caudate lobe and the other extended to the caudate lobe and ventral segments V–VIII), and one central hepatectomy.

**Table 2:** Comparison of demographic data between both groups

	MLLR-RE (*n* = 9)	Ct-MLLR (*n* = 18)	*p* value
Age (years)^a^	67.0 (46–74)	68.5 (56–82)	0.503
Gender (M:F)	6:3	12:6	0.127
Tumor type			
• HCC	3	8	
• CRLM	3	7	
• ICC	3	3	
BMI (kg/m^2^)^a^	25.8 (20.8–36.4)	24.1 (21.2–36.4)	0.395
ASA score^a^	3 (2–4)	3 (2–4)	0.805
• III	8	14	
• IV	1	4	
Cirrhosis/NASH	3 (33.3)	3 (16.7)	0.367
Preoperative tumor size (cm)^a^	6.6 (3–16)	5.1 (1.6–14)	0.129
Prior abdominal surgery	7 (77.8)	8 (44.4)	0.217
IWATE score^a^	10 (8–11)	9 (6–11)	0.135
• 7	0	1	
• 8	1	3	
• 9	1	6	
• 10	4	5	
• 11	3	3	
Hepatectomy (right/central/left)	5:1:3	10:2:6	1.000

Data are expressed as *n* (%) unless otherwise specified

*BMI* body mass index, *ASA* American Society of anesthesiologist, *HCC* Hepatocellular carcinoma, *ICC* Intrahepatic cholangiocarcinoma, *CRLM* Colorectal liver metastasis, *MLLR-RE* major laparoscopic liver resection post radioembolization group, *Ct-MLLR* control major laparoscopic liver resection group, *RE* radioembolization

^a^Values are median (range)

All the resections as graded by IWATE difficulty were advanced (2 cases) or expert (7 cases) levels. The median risk score for adverse intraoperative events was 9 (9–14), meaning high risk. Seven patients (77.8%) had prior abdominal surgery; therefore, careful adhesiolysis was needed in 4 patients (no. 3, no. 4, no. 8, and no. 9) due to severe adhesions to adjacent organs or the abdominal wall. The median operative time was 478 min (328–600) with median pedicle clamping time of 90.5 min (53–133).

**Fig. 3:**
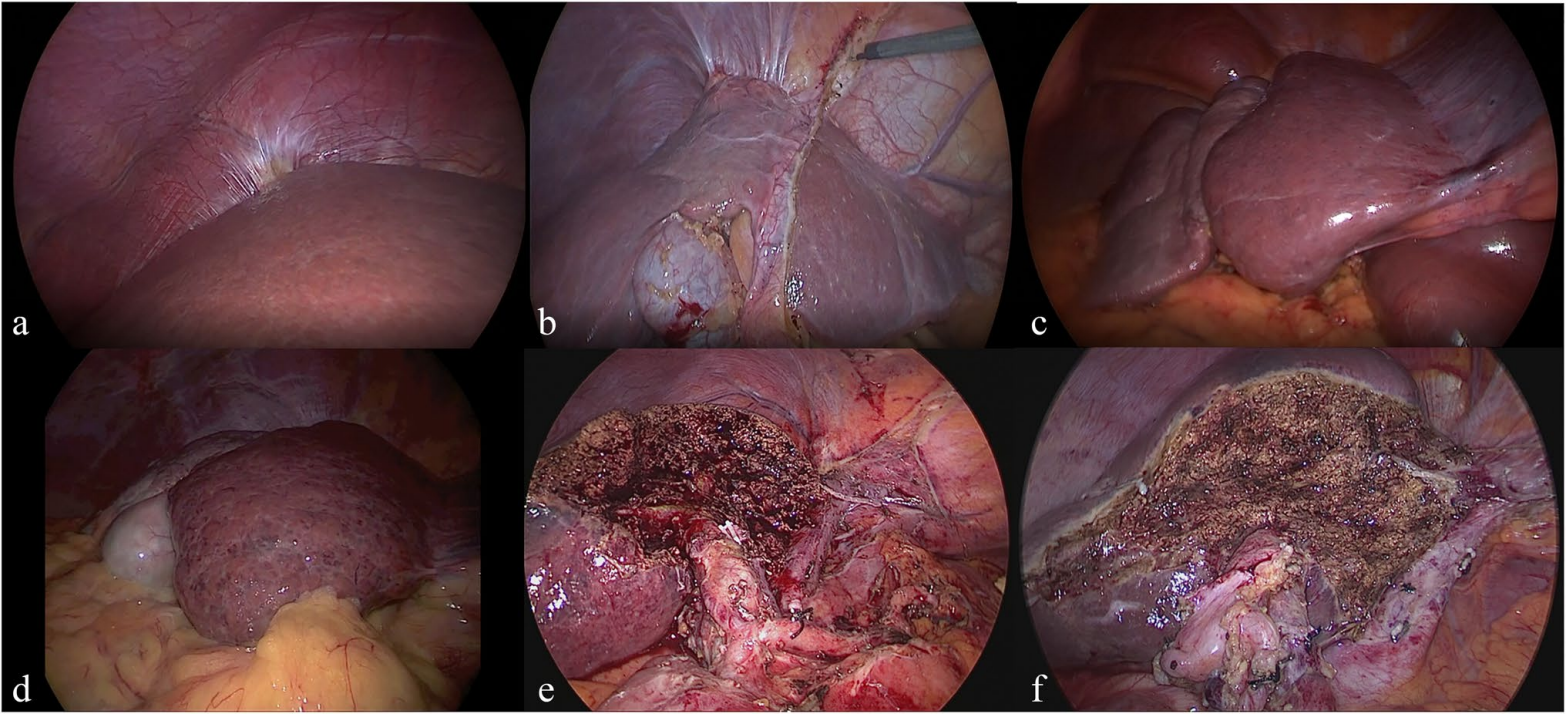
**a** Severe RE-related adhesions across the diaphragm observed during a right hepatectomy with partial right diaphragm resection (patient no. 4 in Table 1). **b** Severe RE-related adhesions next to the confluence of the suprahepatic veins during a central hepatectomy (patient no. 7 in Table 1). **c**, **d** Atrophy of right lobe with marked hypertrophy of the left lobe due to RE in a healthy and cirrhotic liver, respectively (patient no. 2 and 1 in Table 1). **e**, **f** Final view of a left hepatectomy extended to the caudate lobe ventral branches of segment 5 and 8 and lymphadenectomy (patient no. 8 in Table 1) and of a left hepatectomy extended to the caudate lobe and ventral segment V–VIII (patient no. 9 in Table 1)

One intraoperative adverse event took place in patient no. 5: intraoperative bleeding (1000 cc) from a liver tear in a fatty-fragile liver. This was controlled with pedicle-clamping and suture with a barbed wire, with no hemodynamic consequences. Intraoperative transfusion of two units of red blood cells (RBCs) and two units of fresh frozen plasma (FFP) were needed in this patient. No more patients needed RBCs or FFP intra or postoperative transfusion. Figure 3 shows severe RE-related liver adhesions (Fig. 3a, b), an intraoperative view of the atrophy and hypertrophy complex due to RE (Fig. 3c, d), and the final view of both left extended hepatectomies (Fig. 3e, f).

### Short-term outcomes of M-LLR after RE

The median postoperative hospital stay was 3 days (2–11). No PHH occurred but one grade A PHLF was recorded. Two Clavien–Dindo ≥ III complications were recorded. One Clavien–Dindo IIIb complication occurred in patient no. 4 who required an exploratory laparoscopy on POD#1, due to a jejunal perforation, which was solved with abdominal lavage and primary closure. One specific liver complication was recorded. This patient needed readmission due to a biloma that was managed with US-guided drainage and i.v. antibiotic therapy (Clavien–Dindo IIIa). At 90-day follow-up, no deaths were reported.

### Survival and long-term outcomes (MLLR-RE group)

**Fig. 4:**
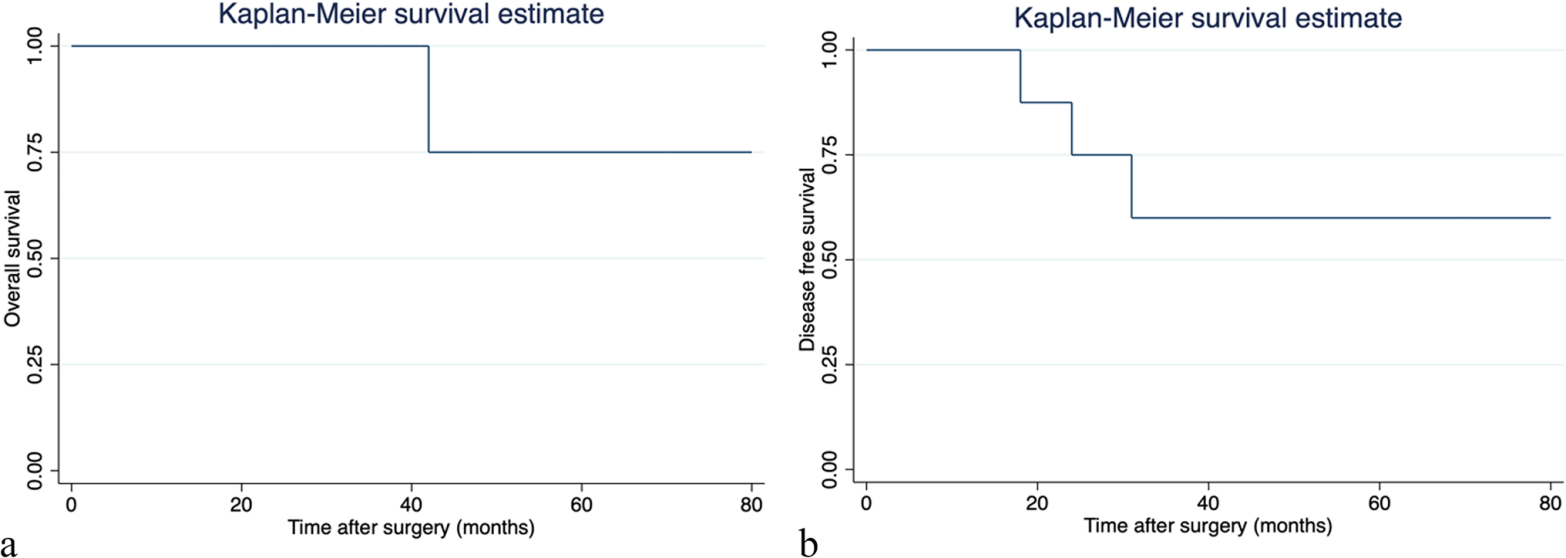
**a** Kaplan–Meier survival curves of overall survival in the laparoscopic major hepatectomy post-RE (MLLR-RE) group. **b** Kaplan–Meier survival curves of disease-free survival in the laparoscopic major hepatectomy post-RE (MLLR-RE) group

With a median follow-up of 44 months (range 6–83 months), three patients presented tumor recurrence (2 CRLM and 1 HCC). Two recurrences were extrahepatic (one in the abdominal wall (HCC) and the other peritoneal (CRLM)). The third was a liver, ovarian, and peritoneal recurrence (CRLM). Median time to recurrence was 18, 24, and 31 months. One-, 3-, and 5-year overall survival was 100%, 100%, and 75% respectively. One-, 3-, and 5-year disease-free survival was 100%, 59%, and 59% respectively. Kaplan–Meier curves are presented in Fig. 4. With the referred follow-up, the only observed death in our series was a patient diagnosed with CRLM, who died 42 months after surgery because of disease progression.

### Pathologic characteristics

Pathologic analysis yielded the result of free margin (R0) in 88.9% (8 patients) of patients and one microscopic (< 1 mm) positive margin (R1). In this patient (case no. 5, Table 1), the real margin was not studied. The section of the hilar plate—including the right biliary branch—was performed with an endostapler. For histological assessment, the 5-mm stapler line was removed with scissors and the studied margin was 5 mm far from the real margin. This patient is currently alive and free of disease after 46 months of follow-up.

Necrosis caused by RE was found in different proportions among the patients (Table 1S, Supplementary Material). Three patients presented < 50% of necrosis (33.3%), the remaining 6 patients exhibited > 50% of necrosis. Among them, 5 patients (55.5%) presented intense necrosis (50–99%) and 1 patient (11.1%) presented complete necrosis (100%) (Table 1S, Supplementary material).

### Matched analysis

The matched groups were comparable as shown in Table 2. The median operative time was longer in the MLLR-RE group, but this did not reach statistical significance (478 vs. 407 min; *p* = 0.135). There were no significant differences in intraoperative blood loss (median 50 vs. 150 ml; *p* = 0.621), RBC transfusion (1 vs. 1; *p* = 1.000), or median pedicle clamping time (90.5 vs. 74 min; *p* = 0.133) between the post-RE and the control group. No significant differences were found in overall complications (4 vs. 6* p* = 0.683) and Clavien–Dindo ≥ III complications (2 vs. 1 *p* = 0.250). One patient in each group presented liver-specific complications, requiring hospital readmission with no significant differences between both groups (1 vs. 1; *p* = 1.000). In both cases, the cause of the liver-related morbidity was an infected biloma that required US-guided drainage and i.v. antibiotic therapy (Clavien–Dindo IIIa). Both patients were discharged after 10 and 5 days, respectively, with no further complications. A comparison of surgical and postoperative outcomes between both groups and with non-matched patients is provided in Table 3 and Table 2S (supplementary material), respectively.

**Table 3:** Intraoperative and postoperative outcomes between both groups

	MLLR-RE (*n* = 9)	Ct-MLLR (*n* = 18)	*p* value
Intraoperative factors			
• Operative time (min)^a^	478 (328–600)	407 (212–576)	0.135
• Pedicle clamping (min)^a^	90.5 (53–133)	74 (36–232)	0.133
• Estimated blood loss (ml)^a^	50 (50–1000)	150 (10–500)	0.621
• Blood transfusion	1 (11.0)	1 (5.6)	1.000
Postoperative outcomes			
• Hospital stay (day)^a^	3 (2–10)	4 (2–15)	0.300
• Overall Clavien–Dindo complications	4 (44.4)	6 (33.3)	0.683
• Clavien–Dindo ≥ III	2 (22.2)	1 (5.6)	0.250
• Specific liver morbidity	1 (11.1)	1 (5.6)	1.000
Hospital readmission	1 (11.1)	1 (5.6)	1.000
90-day mortality	0 (0.0)	0 (0.0)	1.000

Data are expressed as *n* (%) unless otherwise specified

*MLLR-RE* major laparoscopic liver resection post radioembolization group, *Ct-MLLR* control major laparoscopic liver resection group, *RE* radioembolization

^a^Values are median (range)

Median hospital stay was similar between both groups (3 vs. 4 days; *p* = 0.297). At 90-day follow-up, no deaths were reported.

As abovementioned, one microscopic positive margin (R1) in the MLLR-RE group (patient no. 5) was recorded, but in the remaining 8 patients (88.9%) a pathologic free margin (R0) was achieved. Among the Ct-MLLR group, all surgeries were R0. Tumor free-margin rates were not statistically different between both groups (*p* = 0.333).

## Discussion

RE is used as a downstaging or palliative treatment for advanced primary or secondary liver tumors [32–34]. Among patients with HCC and ICC, the previous literature has shown that RE produces clinically significant reductions in tumor size with a consequent downstaging that allows, in some cases, surgical radical therapies [3, 35, 36]. In addition, RE has also shown the capacity of making previously unresectable CRLM [4]. Besides reducing tumor size, lobar RE induces hypertrophy of the contralateral lobe allowing curative surgery in some patients with previously insufficient FLR [5]. RE can arrest tumor growth in more than 90% [1] of patients resulting in disease control rates that range from 75 [37] to 90% [38]. Consequently, RE provides synchronous tumor treatment along with hypertrophy. This quality allows RE to be used as a bridge to resection procedures incorporating a valuable test-of-time before resection that optimizes patient selection and may improve oncological outcomes by minimizing recurrence rates. For complex liver malignancies, the combination of RE and a minimally invasive approach has not been reported as a viable option. Herein, we report this series that supports the use of this combined strategy for major hepatectomies in selected cases with adequate feasibility, safety, and short- and long-term outcomes.

Patients with large primary (HCC or ICC) or extensive secondary tumors (CRLM) usually need major hepatectomies to obtain a curative treatment. Within those patients, insufficient FLR (< 30% or 40% for cirrhotic patients) is the principal obstacle to performing radical surgery. In addition to RE, portal vein embolization (PVE) and liver partition with portal vein ligation for staged hepatectomy (ALPPS) are commonly used strategies to generate hypertrophy before surgery [39]. Open ALPPS and open liver resection (OLR) after RE or PVE had proven suitable surgical and oncological outcomes [12, 40].

The short- and long-term advantages of laparoscopic liver surgery over the open approach have already been established. The laparoscopic approach has proven to achieve significantly lower number and less severe complications along with a shorter hospital stay and a lower dose of morphine [41]. Furthermore, the laparoscopic approach to liver resections has reduced the blood loss and the need for transfusion compared to the open route [42]. As recently demonstrated, postoperative complications were independently associated with decreased overall and disease-free survival after surgery for CRLM with curative intent [43]. The main limitations of laparoscopic liver resections are patients with tumor extension to major vessels requiring complex vascular or hepatobiliary reconstruction. However, these boundaries could be surpassed by experienced minimally invasive hepatobiliary surgeons in specialized centers. However, data regarding minimally invasive (MI) ALPPS or laparoscopic hepatectomy after PVE or RE is still scarce.

A recent systematic review with 27 patients diagnosed with malignant liver tumors that underwent MI-ALPPS has reported 30.8% of Clavien–Dindo ≥ III complications (15.4% IIIa and 15.4% IIIb), a length of stay between 8 and 33 days, and no in-hospital mortality [44]. Okumura et al. [45] published a propensity score matched (PSM) study comparing laparoscopic vs. open two-stage hepatectomy after portal vein ligation (PVL) or PVE among patients with CRLM. Within the PSM laparoscopic group and after second-stage hepatectomy, 6 patients (24%) had Clavien–Dindo ≥ III post-operative complications. Similarly, 6 patients (24%) presented liver-specific Clavien–Dindo ≥ III complications: among these, 2 (8%) and 3 (12%) patients experienced liver failure (≥ ISGLS grade B) and biliary leakage (≥ ISGLS grade B), respectively. One death (4%) was reported within 90 days of surgery. In agreement with these studies, in our series, MLLR after RE appears to be a safe approach with 2 patients (22%) with Clavien–Dindo ≥ III complications, 1 patient (11%) with a liver-specific Clavien–Dindo ≥ III complication, no liver failures, and no 90-day mortalities.

Survival and long-term outcomes in the MLLR-RE group show that 1-, 3-, and 5-year OS was 100%, 100%, and 75% respectively. Disease-free survival at 1, 3, and 5 years was 100%, 59%, and 59% respectively. OS and DFS rates are encouraging suggesting that LLR in patients with unresectable tumors that responded to RE is an excellent option. Nevertheless, these results should be interpreted with caution due to the small sample size and its heterogeneity. In addition, this survival function includes patients with 3 different tumors that present different prognoses. Even so, it seemed interesting to us to represent how patients with a very poor prognosis at diagnosis could obtain some survival benefit by combining RE and laparoscopic liver surgery.

Textbook outcomes (TOs) are a feasible and useful parameter for evaluating the quality of surgical care. TOs in liver surgery (TOLS) have been recently defined [17]. TOLS in LLRs were defined as the absence of intraoperative incidents of grade ≥ 2, postoperative bile leak grades B or C, severe complications (Clavien–Dindo ≥ III), postoperative repeat surgery, readmission within 30 days after discharge, in-hospital mortality, and the presence of an R0 resection margin. In our series, one intraoperative grade ≥ 2 incident was recorded in patient no. 5. The same patient needed readmission and the anatomopathological study revealed a microscopic positive margin (R1). Additionally, patient no. 4 underwent repeat surgery due to a Clavien–Dindo IIIb complication. According to these outcomes, major LLRs after RE in our series seem to fulfill these quality standards in a high proportion: 7 patients (77.8%) in our series achieved TOLS, which compares well with the published standards [46, 47].

Radioembolization was initiated in our center in the early 2000s as one of the first centers in Europe. In the same way, we initiated our experience of post-RE surgery in 2005. Being liver surgery after RE an unexplored scenario, the procedures were performed by an open approach. In the absence of evidence of the results of laparoscopic approach in this setting, we proceeded with caution, being our first laparoscopic case—March 2011—a segmentectomy of segment VI in a cirrhotic patient. The result was satisfactory, and the patient was discharged 3 days after surgery with no complications. As time passed, the team gained experience to face more complex surgeries. If we look at the distribution over time, between 2011 and 2014, 8 liver resections were performed after RE in our center: 6 were open surgery (75%) and 2 laparoscopic (25%) (one of them a major resection). After this first major resection in 2014 (to our knowledge, the first major resection post RE published in the literature [16]), in the next period, this was our preferred approach. From 2015 to 2020, 13 liver resections were performed after RE: 10 of them laparoscopic (77%) and only 3 open (23%). It is convenient to highlight that among the 12 patients that underwent laparoscopic surgery, none required conversion to an open or assisted approach.

After performing the first laparoscopic major hepatectomy in 2014, this was our preferred approach. The only exclusion criteria for laparoscopy was the need for complex vascular reconstruction (portal reconstruction, suprahepatic reconstruction, and a left lateral segmentectomy associated with a thrombectomy). From our point of view, this is an added value of this study since all are non-selected consecutive cases.

As a result of our experience and to ensure good results, in our opinion, 3 main considerations should be highlighted. Firstly, the importance of meticulous patient selection for resection after RE. Extrahepatic disease must be ruled out, and adequate FLR guaranteed, pre-operative state-of-the-art imaging studies and thorough anesthetic evaluations must be performed to ensure the ability of the patient to endure the surgery. All these are key points in the pre-operative evaluation. Secondly, major LLR after RE should be performed by a highly experienced laparoscopic liver surgeon. According to the IWATE score [21], median difficulty of LLRs in this MLLR-RE series was 10 (8–11) and the risk of intra-operative events [23] was 9 (9–14). This represents an expert level of difficulty and a high/extremely high risk of intra-operative complications. For this reason, we warn that M-LLRs in post-RE patients should be performed by HPB surgeons who have completed their laparoscopic learning curve. Finally, the importance of working along with a high-qualified multidisciplinary surgical team with experience in laparoscopic liver surgery is key to obtaining successful results in any complex hepatic procedure.

Several limitations associated with a retrospective analysis should be acknowledged. The encouraging results of this series should not distract us from the fact that the reliability of the conclusions is limited because of the small sample size and unknown impact of selection bias. However, this is the only series to date on MLLR-RE, which confers relevance to this experience. Further studies with larger series are needed to confirm the results herein presented.

## Conclusions

Laparoscopic major liver resections post-RE are feasible and safe. The results of this preliminary series compare well with the current TOLS. Moreover, our matched study shows that, despite the greater technical complexity, perioperative and short-term outcomes of MLLR-RE (in patients diagnosed with HCC, ICC, or CRLM) seem to be comparable with those for patients that undergo MLLR for the same malignancies but without prior RE. The combination of a multidisciplinary evaluation with a precise pre-operative evaluation and a highly experienced laparoscopic hepatobiliary surgery team is mandatory to ensure good outcomes.

## Supplementary Information

Below is the link to the electronic supplementary material.

Supplementary file1 (DOCX 16 KB)

## Data Availability

Not applicable.
