# Supplementary material for: Pure laparoscopic major liver resection after yttrium90 radioembolization: a case-matched series analysis of feasibility and outcomes
Source: Langenbecks Arch Surg. 2022 Feb 28;407(3):1099–111. doi: 10.1007/s00423-022-02474-z (PMC9151566; doi:10.1007/s00423-022-02474-z)
Supplement: Supplementary file 1 — Supplementary file1 (DOCX 16 KB) [file 423_2022_2474_MOESM1_ESM.docx]

SUPPLEMENTARY MATERIAL

| Table 1S. Degree of necrosis of the MLLR-RE group due to radioembolization treatment | | |
| --- | --- | --- |
| Patient | **Tumor type** | **Necrosis (%)** |
| 1 | HCC | 15 |
| 2 | ICC | 30 |
| 3 | CRLM | 70 |
| 4 | HCC | 70 |
| 5 | ICC | 70 |
| 6 | HCC | 15 |
| 7 | CRLM | 70 |
| 8 | ICC | 90 |
| 9 | CRLM | 100 |
| *HCC* Hepatocellular carcinoma*, ICC* Intrahepatic cholangiocarcinoma*, CRLM* Colorectal liver metastasis | | |

| **Table 2S.** Intraoperative and postoperative outcomes between MLLR-RE and the non-matched group | | | |
| --- | --- | --- | --- |
|  | **MLLR-RE (n:9)** | **Non matched**  **(n:83)** | ***p* value** |
| Intraoperative factors |  |  |  |
| - Pedicle clamping (min) ^a^ | 90.5 (53-133) | 67.5 (0-232) | 0.280 |
| - Estimated blood loss (ml) ^a^ | 50 (50-1000) | 90 (10-1000) | 0.210 |
| - Blood Transfusion | 1 (11.0) | 7 (9.0) | 0.834 |
| Postoperative outcomes |  |  |  |
| - Hospital stay (day) ^a^ | 3 (2-10) | 2 (2-21) | 0.830 |
| - Overall Clavien-Dindo complications | 4 (44.4) | 17 (21.5) | 0.126 |
| - Clavien–Dindo ≥ III | 2 (22.2) | 4 (5.1) | 0.053 |
| - Specific liver morbidity | 1 (11.1) | 1 (1.2) | 0.055 |
| Hospital readmission | 1 (11.1) | 4 (5.1) | 0.458 |
| *Data are expressed as n (%) unless otherwise specified*  *MLLR-RE major laparoscopic liver resection post radioembolization group,* *RE radioembolization.*  ^a^ Values are median (range) | | | |
